# Invertebrates and herptiles for livelihoods—ethnozoological use among different ethnic communities in Jammu and Kashmir (Indian Himalayas)

**DOI:** 10.3389/fphar.2022.1043155

**Published:** 2023-01-12

**Authors:** Musheerul Hassan, Shiekh Marifatul Haq, Muhammad Shoaib Amjad, Riyaz Ahmad, Rainer W. Bussmann, José Manuel Pérez de la Lastra

**Affiliations:** ^1^ Clybay Research Private Limited, Bangalore, India; ^2^ Department of Ethnobotany, Institute of Botany, Ilia State University, Tbilisi, Georgia; ^3^ Department of Botany, Women University of Azad Jammu & Kashmir, Bagh, Pakistan; ^4^ Birmingham Institute of Forest Research, University of Birmingham, Birmingham, United Kingdom; ^5^ National Center for Wildlife, Riyadh, Saudi Arabia; ^6^ State Museum for Natural History, Karlsruhe, Germany; ^7^ Biotechnology of Macromolecules Research Group, Instituto de Productos Naturales y Agrobiología (IPNA-CSIC), San Cristóbal dela Laguna, Spain

**Keywords:** cross-culture, ethnozoology, medicinal animals, livelihood, Kashmir

## Abstract

Background: Ethnic communities have relied on animals and their derived products for ages, and their use is often intricately related to many cultural features. In remote regions across the globe, indigenous peoples have been using invertebrates and herptiles for a variety of purposes (medicine, food, culture, and spiritual importance); however, related scientific research is sparse, particularly in the western Himalayas. In this respect, we collected useful information on invertebrates and herpetofauna from Jammu and Kashmir, India, across different ethnic groups, i.e., Gujjar, Bakarwal, Dogra, Kashmiri, and Pahari.

Methodology: The data were gathered using semi-structured interviews followed by group discussions. The information gathered was analyzed using ordination techniques (principal component analysis). The Venn diagram was used to investigate cross-cultural similarities and differences between ethnic groups.

Results: We documented 30 species belonging to five classes and 20 families used for different ethnozoological practices (medicinal, magico-religious, food, costume, omen, poultry, and agricultural purposes). The use of fauna resources varied across ethnic groups, and cross-cultural examination revealed that Kashmiri and Pahari populations were more similar in their species utilization. The maximum number of species (27%) was uniquely used by Kashmiri, followed by Pahari (17%), and the least by Dogra and Gujjar (3% each). The ethnozoological use of all documented species is unprecedented. In addition to ethnozoological usage, various documented species (*Apis cerana*, *Apis mellifera*, *Hirudinaria granulosa*, and *Bombyx mori*) were also important for the local population’s livelihoods.

Conclusion: Our findings can be considered the baseline for understanding the relationship of invertebrates and herptiles with specific ethnic groups and will aid in the development of future research projects that can assess the interaction between local fauna and the diverse ethnic groups.

## 1 Introduction

Merging ethnic knowledge with scientific approaches can help implement tenable use of natural resources to the benefit of communities (Roux et al., 2006). Despite the fact that conventional therapeutics are mostly centered on plant resources and their derived materials, fauna are also a vital part of traditional medicine in different cultures (Loko et al., 2019; Haq et al., 2020). Both invertebrates and herptiles are used in treating health problems and are also employed in religious ceremonies and magic (Costa-NetoEntomotherapy, 2005; Altaf et al., 2020). In a variety of ethnic communities, people use different species of herptiles and invertebrates in unique ways (Chellappandian et al., 2014). Traditional knowledge as such is a vital aspect of cultural heritage that can present the association between ethnic communities and nature (Alves et al., 2013). Such traditional endemic knowledge encompasses ethnozoology (Anderson et al., 2012). Ethnozoological knowledge collected from local people can help in identifying new bioresources with commercial value, especially in food and medicine (Malmfors et al., 2002). The use of fauna species to treat health problems (zootherapy) has long been practiced across the globe. In China, it has been reported that earthworms were used to treat diseases almost 4,000 years ago (Chee and Mao, 2021). According to Alves and Rosa (2005), over 5,000 animal species are part of traditional Chinese medicine (TCM). Similarly, 15–20% of Ayurvedic medicine includes animals and their products (Smruti, 2021) with at least 500 species of invertebrates used to treat a variety of health disorders (Prakash and Verma, 2021). Many insect species are used alive, cooked, ground, and made into infusions, plasters, and ointments for curative and preventive medicine (Costa-NetoEntomotherapy, 2005; Aziz et al., 2018). Currently, it is estimated that 8.7% of the important compounds used in modern medicine are obtained from animals or based on their derivatives (Altaf et al., 2020). In developing countries such as India, Pakistan, and Bangladesh, traditional medicine is commonly seen as another source for primary healthcare, while in the developed world, we observe a continuously growing tendency toward the usage of traditional medicine. Meanwhile, the documentation of indigenous traditional knowledge has become imperative due to profound changes in the culture and socioeconomic profile of local communities around the globe (Alves et al., 2010; Vijayakumar et al., 2015; Hamid et al., 2021).

India possesses a highly diverse fauna, and it is estimated that 10% of global reptile species (including Squamata and Testudines), amphibians, and insects are found here (Dar and Khuroo, 2020). Despite this enormous diversity, studies on the ethnousage of fauna species are limited, particularly in the Himalayas and Jammu and Kashmir (J&K) (Alves et al., 2010). J&K is a union territory in the northern Himalayan region of India. The region has a rich cultural ethnicity; communities like Kashmiri, Gujjar, Pahari, Dogra, and Bakarwal have been inhabitants for centuries (Hamid et al., 2021). Due to its unique location and climatic conditions, J&K harbors 16% of India’s reptiles, mammals, and invertebrates (Dar and Khuroo, 2020). The present study aimed to understand and document the local knowledge of fauna species across different ethnic groups in Jammu and Kashmir which in turn can help protect this tremendous knowledge from getting lost due to a lack of transmission to the next generation. In this regard, authors have been working for many years and have published many studies (Hutt et al., 1994; Downie et al., 2016; Haq et al., 2019; Hassan et al., 2021), strengthening the gray literature; hence, the said knowledge can be used for future prospects.

Our study will not only assist in understanding the cross-cultural usage of the fauna in the region but can also help to formulate strong incentives for local people to acknowledge and nurture their traditional knowledge and to receive benefits from its continued sustainable use, for example, through targeted development programs. The present study focused on the following objectives: 1) to document ethnozoological uses of invertebrates and herptiles across different ethnic groups in J&K and 2) to analyze (cross-cultural analysis) the use of documented species across the different cultures.

## 2 Materials and methods

### 2.1. Ethnography and socioeconomic potential

**FIGURE 1:**
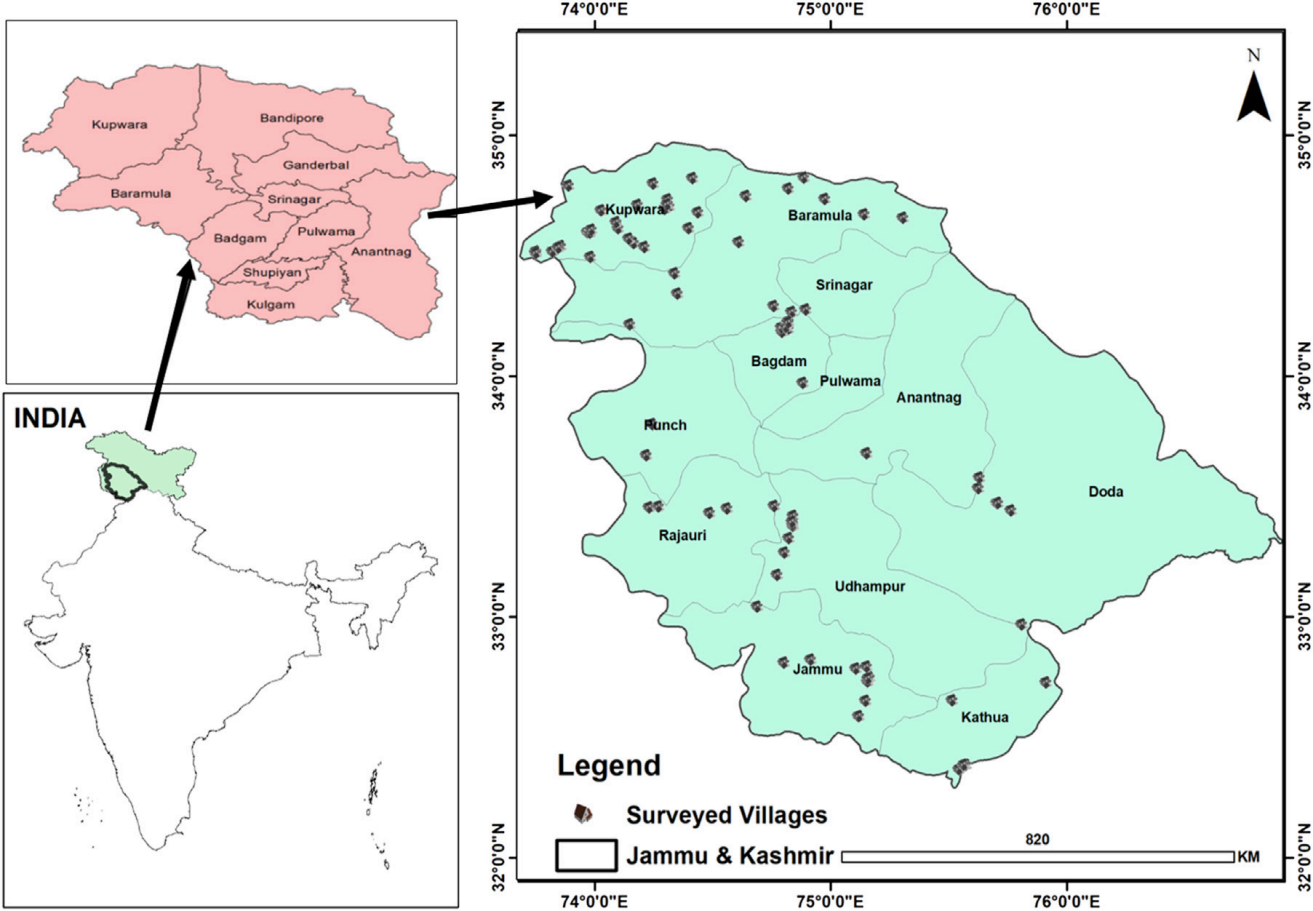
Map of Jammu and Kashmir, India; points show survey sites.

The union territory of Jammu and Kashmir (J&K) (Figure 1) is a north-western Himalayan region in India. The region includes two divisions (Jammu and Kashmir), where Jammu is more diverse, ranging from subtropical plains with hot summers (42°C) and cool winters (13°C) with a monsoonal climate to a more temperate climate in the uplands (1,000 ft), and on the other hand, Kashmir generally has a temperate climate with a maximum of 34°C in summer and a minimum of −9°C in winters. J&K harbors a unique forest system (subtropical dry evergreen, subtropical broad-leaved, subtropical pine, Himalayan moist temperate, Himalayan dry temperate, and subalpine) and vegetation pattern (coniferous and deciduous) with variegated microclimates (Haq et al., 2019; Haq et al., 2022). J&K is an important part of the Himalayas, rich in biodiversity, and rightly recognized as a global biodiversity hotspot (Dar and Khuroo, 2020). Different ethnic communities such as Dogra, Kashmiri, Gujjar, Pahari, and Bakarwal have been living here for centuries, representing a rich cultural diversity (Hassan et al., 2021). Languages spoken by these communities include Dogri, Kashmiri, Phari, and Gujjari. Gujjari is spoken by both the Gujjar and Bakarwal communities (Hassan et al., 2021). Apart from the native languages, Urdu is a common language spoken by all ethnic groups. It is believed that Kashmiri trace their origin from the Indo-European ethnolinguistic group (Downie et al., 2016), Pahari are decedents of the Kash Empire (Hutt et al., 1994), the Ikshvaku (Solar) dynasty of northern India are believed to be the ancestors of the Dogra (Kaur, 2020), and the Gujjar and Bakarwal have migrated from another state (Rajasthan) of India (Tufail, 2014). As per the latest census (2011), the total human population is about 13.6 million, among whom Muslims are the dominant religious group (67%), followed by Hindus (30%), Sikhs (2%), and Buddhists (1%) (http://ecostatjk.nic.in/Digest1314/1%20area%20and%20papulation.pdf). In J&K, the economy of the people is associated with agriculture. Most inhabitants are professional farmers, some are government employees, and many are daily-wage laborers. Bakarwal and Gujjar are especially dependent on livestock, whereas Dogra, Pahari, and Kashmiri rely on agriculture. Traditional medicine is often practiced by specialists, locally called *Hakeems*.

### 2.2. Informant selection and ethnozoological data collection

To document the ethnozoological knowledge, regular field surveys were conducted during 2021. The first author of the study visited different sites with local volunteers (24 times). The whole team interacted with the local people and briefed them about the purpose of the study. Before data collection, we ensured that every informant gave written prior informed consent and that the ISE Code of Ethics was closely followed (Sorensen, 1948; Greig-Smith, 1983; Laird and S.A., 2010). To gather data at all selected sites (Figure 1), we applied a snowball technique, which used semi-structured interviews, meetings, and group discussions (Haq et al., 2020). We selected 153 respondents, of which 87 were men and 66 were women of different age groups (Table 1). The selected respondents were categorized into different professional groups: farmers, shepherds, street vendors, housewives, craftsmen, and shopkeepers following different religions (Islam, Hinduism, and Sikhism). The livelihood sources include agriculture, horticulture, and pastoralism. Young and unmarried women were, however, not allowed to participate in the study due to cultural limitations. Local languages were used for the interview to ensure proper communication. To remove errors and omissions from the obtained data, those data were redisplayed to the respective participants.

**TABLE 1:** Demographic status of the respondents from Jammu and Kashmir, the western Himalayas, India.

Demographic features	Total	Biogeographic region		Ethnic (linguistic) groups				
		Jammu	Kashmir	Kashmiri	Pahari	Bakarwal	Gujjar	Dogra
**Respondents**	153	63 (41.17%)	90 (58.82%)	32	31	33	34	23
**Gender**								
Male	87 (56.86%)	29	58	21	16	21	19	10
Female	66 (43.13%)	34	32	11	15	12	15	13
**Original language**	-	-	-	Kashmiri	Pahari	Gujjari	Gujjari	Dogri
Age Range				27–75	27–75	27–75	27–75	27–75
**Religion**	-	Islam Hinduism Sikhism	Islam Hinduism	Islam Sikhism	Islam Hinduism	Islam	Islam Hinduism	Hinduism
**Profession**	-	-	-	Farmers	Farmers	Shepherd Housewives	Shepherd	Farmers
				Shepherd	Shepherd		Housewives	Shepherd
				Street vendors	Housewives		Craftsmen	Street vendors
				Housewives	Craftsmen		Shopkeepers	Housewives
				Craftsmen	Shopkeepers			Craftsmen
				Shopkeepers				Shopkeepers
**Livelihood source**	-	-	-	AgricultureHorticulture	Agriculture	Pastoralism	Agriculture	HorticultureAgriculture

The selected respondents were asked about the local usage and names of invertebrates and herpetofauna. The corresponding photographs for each species (N = 30) were then identified using field guides and secondary sources (Greig-Smith, 1983; Phillips, 1994; Kakati et al., 2006; Oksanen et al., 2013; Team, 2013; Altaf et al., 2018; Mozhui et al., 2021; Mussarat et al., 2021; Haq et al., 2022) with the help of a local taxonomist from the zoology department at Baba Ghulam Shah Badshah University in Rajouri, J&K, India. Taxonomic verification was also carried out by using the online databases “Integrated Taxonomic Information System” (https://www.itis.gov) and IUCN’s Red List of Threatened Species (https://www.iucnredlist.org).

In order to confirm the species identification for the taxa mentioned with specific ethnousage and vernacular names, the respondents were again shown the collected images. The respondents immediately identified the same taxa each time with ethnousage and vernacular names. The traditional healers (Hakeems) were also shown the verified images in order to avoid any bias and were provided with final verification.

### 2.3. Data analysis

To determine the significance of a particular species in contrast to other species, we employed the use value (Haq et al., 2020) using the formula:

UV=∑U/N,

where “U” is the number of use reports for a particular species and “N” is the total number of respondents.

To analyze the clustering of faunal species among ethnic groups, principal component analysis (PCA) was used by employing the package vegan (Oksanen et al., 2013) in software R ver. 4.0.0 (Team, 2013). A Venn diagram was used to investigate cross-cultural similarities and differences between ethnic groups using Bioinformatics & Evolutionary Genomics software—Venn diagram (http:bioinformatics.psb.ugent.becgi-binlisteVenncalculate_venn.htpl) (Phillips, 1994).

## 3 Results and discussions

### 3.1. Ethnozoological inventory

The current study identified 30 species of herptiles and invertebrates used by local people in two biogeographic regions (Jammu and Kashmir), broadly classified into five classes, namely, Insecta (N = 17), Arachnida (N = 2), Clitellata (N = 3), Amphibia (N = 3), and Reptilia (N = 5) (Table 2). Our sample size was larger than those of earlier fragmented ethnozoological studies from the nearby Himalayan region. For example, Altaf et al. (2018) reported two species of invertebrates, five species of reptiles, and two species of amphibians from Punjab, Pakistan; Mussarat et al. (2021) reported eight invertebrates and two reptiles from Khyber Pakhtunkhwa, Pakistan, implying that people in J&K in the western Himalaya region use more fauna resources for ethnozoological purposes. The high use of insects can be ascribed to the beliefs in the local traditional medical system that prioritizes insects over other classes. The species were further classified into 20 families: Apidae (N = 5) was the dominant family, followed by Formicidae (N = 3), Dicroglossidae (N = 3), Araneidae (N = 2), and Viperidae (N = 2). The detailed inventory including zoological name, local name, family, ethnic groups, part used, and ethnozoological and ethnomedicinal uses is provided in Table 2.

**TABLE 2:** Ethnozoological inventory of documented species from Jammu and Kashmir.

S. No		Scientific name (family) abbreviation	Local name	Parts used	Biogeographic regions		Local usage	Zootherapy (ethnomedicinal profile)	Ethnic groups			Use value	Use reports
					Jam	Kas							
Insecta													
1		*Prionopelta kraepelini* (Forel, 1905) (Formicidae) Pri.kra	Chentee (P)	Whole body	Y	N	Ants are kept in a pot placed in front of patients suffering from spiritual disease and then both are enchanted for some time (P)	Ants are dried, powdered, and mixed with powdered eggshells (*Anser anser domesticus*) and given in small quantities with milk to overcome infertility issues in males (P)	Pahari			0.13	21
2		*Tapinoma melanocephalum* (Fabricius, 1793) (Formicidae) Tap.mel	Chentee (P) Dav Rai (K)	Whole body	Y	Y	----------------------	Ants are ground to make paste, mixed with honey, and applied on sun-burned skin for an hour then washed with turmeric water (P)	Pahari Kashmiri			0.11	18
								Ants are also used to treat erectile dysfunction by mixing the dried, powdered ants with walnuts, almond, and black seeds (^a^)					
3		*Stenamma* kashmirense (Baroni Urbani, 1977) (Formicidae) Ste.kas	Kash Rai (K)	Whole body	N	Y	----------------------	Dried ants are powdered and given with milk to treat impotence (K)	Kashmiri			0.11	17
4		*Junonia* orithya (Linnaeus, 1758) (Nymphalidae) Jun.ori	Panpompree (G, P) Poompar (K)	Whole body	Y	Y	Children used to play with them in fields. (^a^) People also have the belief that if large blue butterflies come often in the home garden, it is a sign of something good happening (K)	----------------------	Pahari Gujar Kashmiri			0.12	19
5		*Bombyx mori* (Linnaeus, 1758) (Bombycidae) Bom.mor	Guachkum (K) Raishm (P)	Cocoons	Y	Y	Silk is obtained and used for costume making, providing livelihood to different people (^a^)	Dried silkworms are powered and taken orally to treat flatulence (P)	Kashmiri Pahari			0.33	51
6		*Plecoptera reflexa* (Guenée, 1852) (Noctuidae) Ple.ref	Neelii (D)	Whole body	Y	Y	Different magicians believe that it is a virtual form of demons (D)	------------------------------------	•Dogra			•0.1	•16
7		*Apis cerana* (Fabricius, 1793) (Apidae) (Api.cer)	Manchar (K)	Honey	Y	Y	Honey is used as food. (^a^) In Islam, honey is treated like an elixir as it has been described as a source of healing in the holy book of Quran (^a^)	Honey is taken orally with lukewarm water to treat cough, cold, and fatigue (^a^)	Pahari			0.4	62
			Madoo (G)	Honeycomb				Honeycomb is taken orally to treat intestinal infections and to glow face skin (K)	Gujjar				
			Maduu (P)	Bee venom				Bee venom is applied topically to treat neuropathic pain and arthritis (K)	Kashmir				
			Madokmi (D)						Dogra				
8		*Apis mellifera* (Linnaeus, 1758) (Apidae) Api.mel	Manchar (*)	Honey	Y	Y	Honey is used as food (^a^)	Honey is taken orally with lukewarm water and lemon to treat cough, cold, fatigue, and skin issues; lose belly fat; treat indigestion and blood pressure issues; and enhance immunity (^a^)	Pahari			0.35	55
			Madoo (G)	Honeycomb				Honeycomb is taken orally to treat intestinal infections and to glow face skin (K)	Gujjar				
			Maduu (P)	Bee venom				Bee venom is applied topically to treat neuropathic pain and arthritis (K)	Bakarwal				
			Madomaki (D,B)						Kashmiri Dogra				
9		*Bombus cornutus* (Frison 1933) (Apidae) Bom.cor	Madoo (G)	Honey	N	Y	Honey is used as food (G)	Honey is mixed with milk, and this cream is applied on skin to overcome skin problems like sun burns and wrinkles. (G)	Gujjar			0.2	32
10		*Apis dorsata* (Fabricius, 1793) (Apidae) Api.dor	Manchar (K)	Honey	Y	Y	Honey is used as food (^a^)	Honey is used as such or with milk or lukewarm water to treat cough, cold, and aging (^a^)	Pahari			0.26	41
			Madoo (G)						Gujjar				
			Maduu (P)						Bakarwal				
			Madomaki (D, B)						Kashmiri Dogra				
11		*Apis florea* (Fabricius, 1787) (Apidae) Api.flo	Maanchar (K)	Honey	N	Y	Honey is used as food (K)	Honey is consumed with duck eggs and walnuts to treat impotence (K)	Kashmiri			0.28	44
12		*Oxya japonica* (Thunberg, 1815) (Acrididae) Oxy.jap	Haalov (K)	Whole body	N	Y	People collect the species and feed them to poultry (K)	----------------------	Kashmiri			0.11	18
13		Aeshna mixta (Latreille, 1805) (Aeshnidae) Aes.mix	Harma zain zain (K)	Wings	N	Y	----------------------	Wings are dried, powdered, and mixed with honey and nuts to treat sexual problems (^a^)	Pahari			0.15	23
			Jahaj (B)						Bakarwal				
			Har (P)						Kashmiri				
14		*Pantala flavescens* (Fabricius, 1798) (Libellulidae) *Pan*.fla	Zainain (P)	Wings	N	Y	----------------------	Wings are dried, powdered, and mixed with honey and nuts to treat sexual problems (P)	Pahari			0.12	19
15		*Pediculus humanus capitis* (De Geer, 1778) (Pediculidae) Ped.hum.cap	Joon (P)	Whole body	Y	Y	----------------------	It is believed that the louse sucks blood from the head which in turn treats brain tumors (^a^)	Gujjar			0.18	28
			Joovaan (G)						Pahari				
			Zovaan (B)						Bakarwal				
			Zoon (D)						Dogra				
16		*Cicadatra acberi* (Distant, 1888) (Cicadidae) Neo.can	Cheen (K)	Sound	N	Y	----------------------	To overcome anxiety, it is advised to go into the lands in the evening and focus on the sound (K)	Kashmiri			0.13	21
17		*Anopheles* lindesayi (Giles, 1900) (Culicidae) Ano.lin	Mooh (K)	Whole body	Y	Y	It is believed that mosquitoes help in paddy ripening (K)	----------------------	Kashmiri			0.12	19
Arachnida													
1		*Neoscona theisi* (Walckenaer, 1841) (Araneidae) Neo.the	Zorul (B)	Web Whole body	N	Y	Webs woven inside homes are treated as a sign of upcoming misfortune. (^a^) The spider is treated sacred in Islam, and it is believed that it has protected the prophet Mohammad from enemies by covering the mouth of cave (^a^)	Webs are placed on the minor cuts to stop bleeding. (^a^)	Gujjar			0.26	40
			Zalur (^a^)						Pahari				
			Makdi (G)						Bakarwal				
			Garala (P)						Kashmiri				
2		*Araneus trifolium* (Hentz, 1847) (Araneidae) *Ara*.tri	Makdi (B)	Web Whole body	Y	Y	Webs woven inside homes are treated as a sign of upcoming misfortune (B)	----------------------	Bakarwal			0.18	28
Clitellata													
1	*Hirudinaria granulosa* (Linnaeus, 1758) (Hirudinidae) Hir.gra		Draik (^a^)	Whole body	Y	Y	----------------------	Used for bloodletting to treat swelling, bruises, and pain. (^a^)		Bakarwal	0.25		•39
										Pahari			
										Kashmiri			
2	*Aporrectodea caliginosa* (Savigny, 1826) (Lumbricidae) Apo.cal		Bumsum (K)	•Whole body	•Y	•Y	A large pile of dung by adding some fertilizers is covered with cloth and kept undisturbed for months, resulting in the development of earthworms. These worms along with dung are added to the soil to help in the soil enrichment and aeration (P)	----------------------		Pahari	0.22		35
			Boosam (P)										
3	*Drawida japonica* (Michaelsen, 1892) (Moniligastridae) Dra.jap		Boosim (P)	Whole body	N	Y	A large pile of dung with some added fertilizers is covered with cloth and kept undisturbed for months, resulting in the development of earthworms. These worms along with dung are added to the soil to help in the soil enrichment and aeration (P)	----------------------		Pahari	0.11		17
Amphibia													
1		*Euphlyctis cyanophlyctis* (Schneider, 1799) (Dicroglossidae) Eup.cya	Meingood (K)	Skin Whole body	Y	Y	Magicians use whole body in their magical practices (K)	Skin is powdered and mixed with seven different spring waters and applied topically to treat skin allergy (^a^)	Gujjar			0.22	35
			Maindak (P)						Pahari				
			Maingad (G)						Kashmiri				
2		*Hoplobatrachus tigerinus* (Daudin, 1803) (Dicroglossidae) Hop.tig	Maindak (P)	Fat Whole body	Y	N	Magicians use whole body in their magical practices (P)	Fat is applied topically to treat headache, muscular pain, and joint pain (P)	Pahari			0.24	38
3		*Fejervarya limnocharis* (Gravenhorst, 1829) (Dicroglossidae) Fej.lim	Khren Mein (K)	Fat	N	Y	----------------------	Fat is applied topically to treat back pain in new mothers (K)	Kashmiri			0.18	29
Reptilia													
1		*Pangshura tecta* (Gray, 1830) (Geoemydidae) *Pan*.tec	Qachve (K)	Shell	N	Y	The shell is kept inside the home to increase wealth. (K) In Hinduism, it is believed to be the vehicle of Lord Vishnu (sustainer of the world) (K)	Fat is applied topically to treat arthritis, epilepsy, and leprosy, and bile is used to treat strangulation (K)	Kashmiri			0.11	18
				Fat									
				Bile									
2		*Platyceps ventromaculatus* (Gray, 1834) (Colubridae) Pla.ven	Sap (G)	Whole body Skin	Y	Y	Skin is used by spiritual healers instead of paper (G)	The snake is beaten to death and kept under a wooden basket above which children (who cannot stand or cannot speak properly) are bathed to overcome the health issue (G)	Gujjar			0.16	25
3		*Daboia russelii* (Shaw & Nodder, 1797) (Viperidae) Dab.rus	Motiguns (P)	Whole body Fat	N	Y	Magicians use whole body in their magical practices (P). The skin is used instead of paper for making amulets to get rid of the demonic possessions (P)	Fat obtained is applied on external male genitalia to increase libido (P)	Pahari			0.18	28
4		*Naja naja* (Linnaeus, 1758) (Elapidae) Naj.naj	Lamba sap (G)	Whole body Skin	Y	Y	The snake is treated as sacred in Hinduism and is believed to be present around the neck of Lord Shiva (^a^)	Skin is powdered and applied topically to treat leprosy (^a^)	Gujjar Pahari			0.24	37
			Ankvalsanp (P)										
			Naag (D)										
5		*Macrovipera lebetinus* (Linnaeus, 1758) (Viperidae) Mac.leb	Khutgunus (K)	Whole body	N	Y	The snake is killed, enchanted, and eaten for 40 days to gain magical powers (K)	----------------	Kashmiri			0.13	21

a depicts local names and local uses by all ethnic groups presented in the column (ethnic group).

G: (Gujjar), Ba: (Bakarwal), P: (Pahari), K: (Kashmiri), and D: (Dogra)—these letters represent the ethnic groups in the respective columns.

Jam (Jammu), Kas (Kashmir), Y: (presence of species in the region), and N: (absence of species in the region).

**FIGURE 2:**
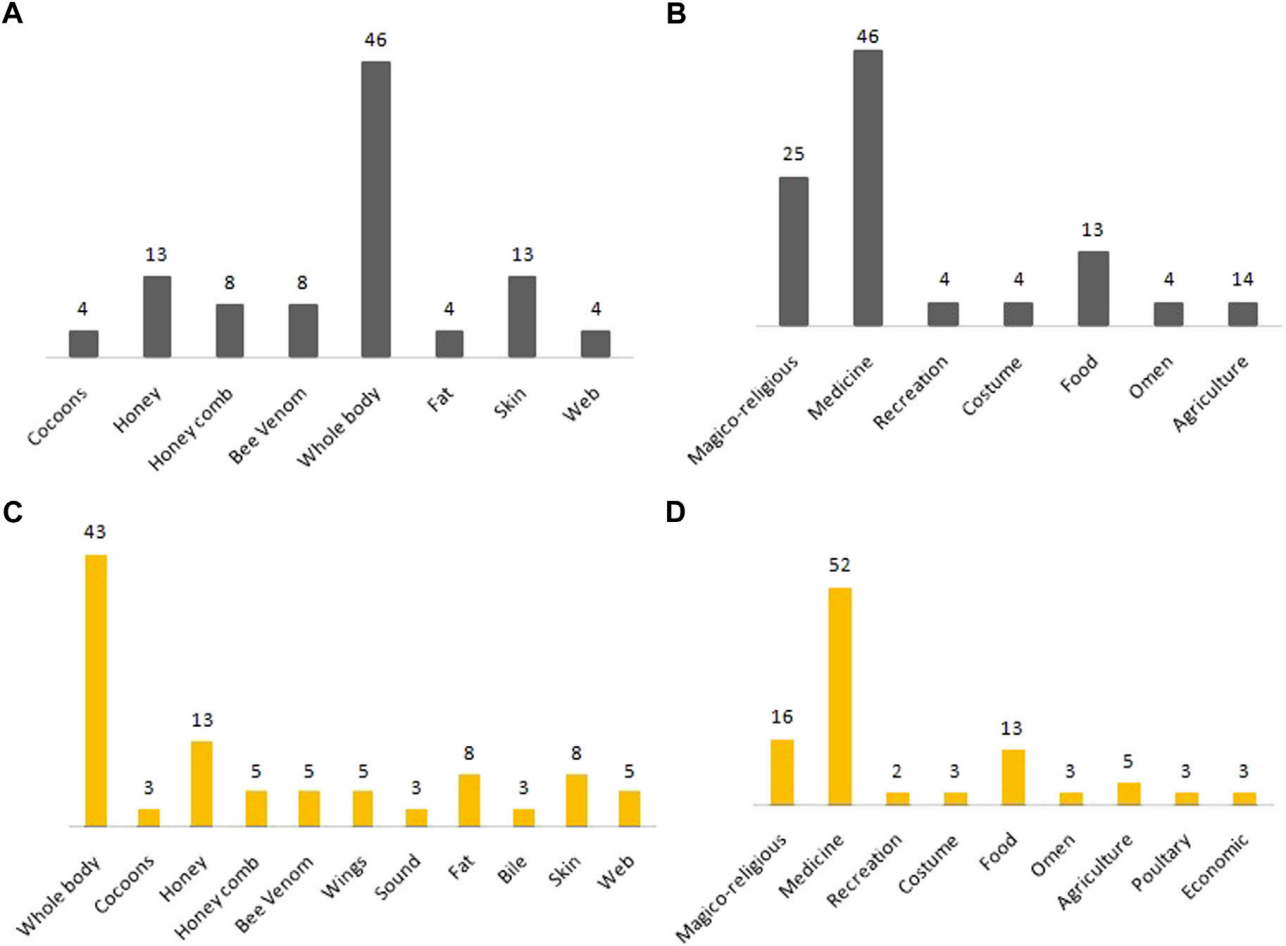
Percentage of used parts and ethnozoological practices of documented species in Jammu **(A,B)** and Kashmir **(C,D)**.

Our results displayed considerable disparities in the proportional use of fauna species among the two biogeographic regions. Among all documented species (N = 30), only 17 were recorded in the Jammu region, out of which 10 belonged to Insecta, two to Amphibians, two to Clitellata, two to Reptilia, and one to Arachnida (Table 2). The different body parts used for different ethnozoological usage included the whole body (46%), skin (13%), and honey (13%) (Figure 2A). Kakati et al. (2006) reported the ethnousage of different body parts such as skin, fat, flesh, and bile from India. Similarly, Mozhui et al. (2021) also reported the usage of different body parts for traditional usage. In the present study, the most important ethnozoological uses were medicinal (46%), followed by magico-religious (25%), food (13%), and costume (12%) (Figure 2B). Our findings were consistent with the results of Altaf et al. (2020), who reported the ascendency of medicinal and magico-religious usage of different amphibians, reptiles, and invertebrates from the Pakistan Himalayas.

In contrast to Jammu, across Kashmir, we recorded 28 species of which 16 were Insecta, five were Reptilia, three were Clitellata, three were Arachnida, and two were Amphibians (Table 2). The different body parts used included the whole body (43%), honey (13%), and skin (8%) (Figure 2C). The recorded ethnozoological uses included medicinal (52%), magico-religious (16%), and food (13%) (Figure 2D). Gogoi and Bora (2020) reported the ethnozoological usage of different body parts from Assam, India. Similarly, Vijayakumar et al. (2015) reported a variety of body parts used for medicinal, magico-religious, and food usage from Kerala, India. The common use of species in the Kashmir region for ethnozoological practices can be ascribed to social, economic, cultural, and religious factors; also, people in the region have a strong faith in traditional medicine.

### 3.2. Ethnomedicinal profile

**FIGURE 3:**
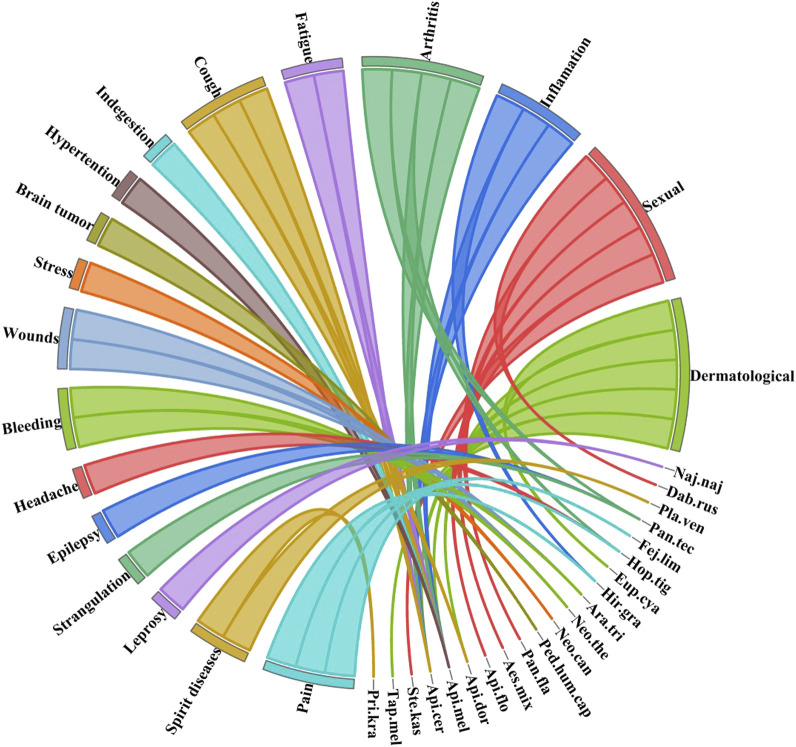
Number of diseases treated by documented species. The direction of the lines shows species association with the particular disease, and the thickness of each bar shows the number of species used to treat that particular disease. The complete name of each species is shown in Table 2.

In the present study, we documented a total of 18 diseases (dermatological issues, sexual issues, inflammation, arthritis, fatigue, cough, indigestion, hypertension, brain tumors, stress, wounds, bleeding, headache, epilepsy, strangulation, leprosy, spirit diseases, and pain) treated with the documented fauna. Among these, dermatological issues were treated with the highest number of species (N = 5) (*Euphlyctis cyanophlyctis*, *Apis dorsata*, *Apis mellifera*, *Apis cerana*, and *Tapinoma melanocephalum*). Similarly, sexual issues were also treated using five species (*Stenamma kashmirense*, *Apis florea*, *Aeshna mixta*, *Pantala flavescens*, and *Daboia russelii*)*.* A complete inventory can be found in Table 2; Figure 3. The use of these species for the aforementioned diseases can be attributed to the traditional knowledge of the local people and their faith in the traditional medicinal system. Bagde (2014) reported the use of *Apis dorsata*, *Apis mellifera*, *Apis cerana* for conjunctivitis, *Hirudinari granulosa* for ulcers, *Aporrectodea caliginosa* for delectation, the cocoon of *Bombyx mori* for pneumonia, and the web of *Neoscona theisi* for bleeding from Madhya Pradesh, India. Jamir and Lal (2005) reported the use of the skin and fat of *Euphlyctis cyanophlyctis* for dermatological and rheumatic pains, respectively. Das (2015) reported the use of different invertebrates for the treatment of cough and asthma from northeast India.

### 3.3. Hirudotherapy and maintenance of leeches

In medieval medicine, the medicinal leech was used to extract blood from patients as a part of a process to balance the humors (Wells et al., 1993). In modern medicine, leech therapy has been used for microsurgery to stimulate circulation to salvage skin grafts (Sig, Guney, Guclu, Ozmen). A national survey (Grau et al., 2018) disclosed that about 70% of French hospitals use leech therapy, clearly revealing the medicinal potential of leeches in the modern world. In this study, we found that ethnic people used leech therapy for the treatment of inflammation, bruises, and pain. Only few people, locally called “drikvale,” have performed hirudotherapy (drikeileaj) for generations. Apart from the medicinal value, the local people have a respectful attitude toward the leeches due to their curative attribution. It is important to mention that the medicinal leech (*Hirudo medicinalis*) (photo plate 1a) is not found in Kashmir, and people associated with hirudotherapy purchase it from other parts of the country (India). Leeches are properly maintained and monitored to avoid any infection or disease. A clean clay pot, locally called *Nooat* or *Matka* (photo plate 1b), half filled with clean mountain or spring water, is used to keep the leeches. A change of water is carried out once a week, the temperature is maintained between +4°C and +20°C, and a dark place is chosen as the storage place. These are all requirements that in fact meet RICARIMPEX recommendations (RICARIMPEX SAS-Eysines, an exclusive supplier of leeches in France and international leader, FDA approved, recommends conditions of storage of leeches) (Grau et al., 2018).

### 3.4. Cross-cultural use of species

**FIGURE 4:**
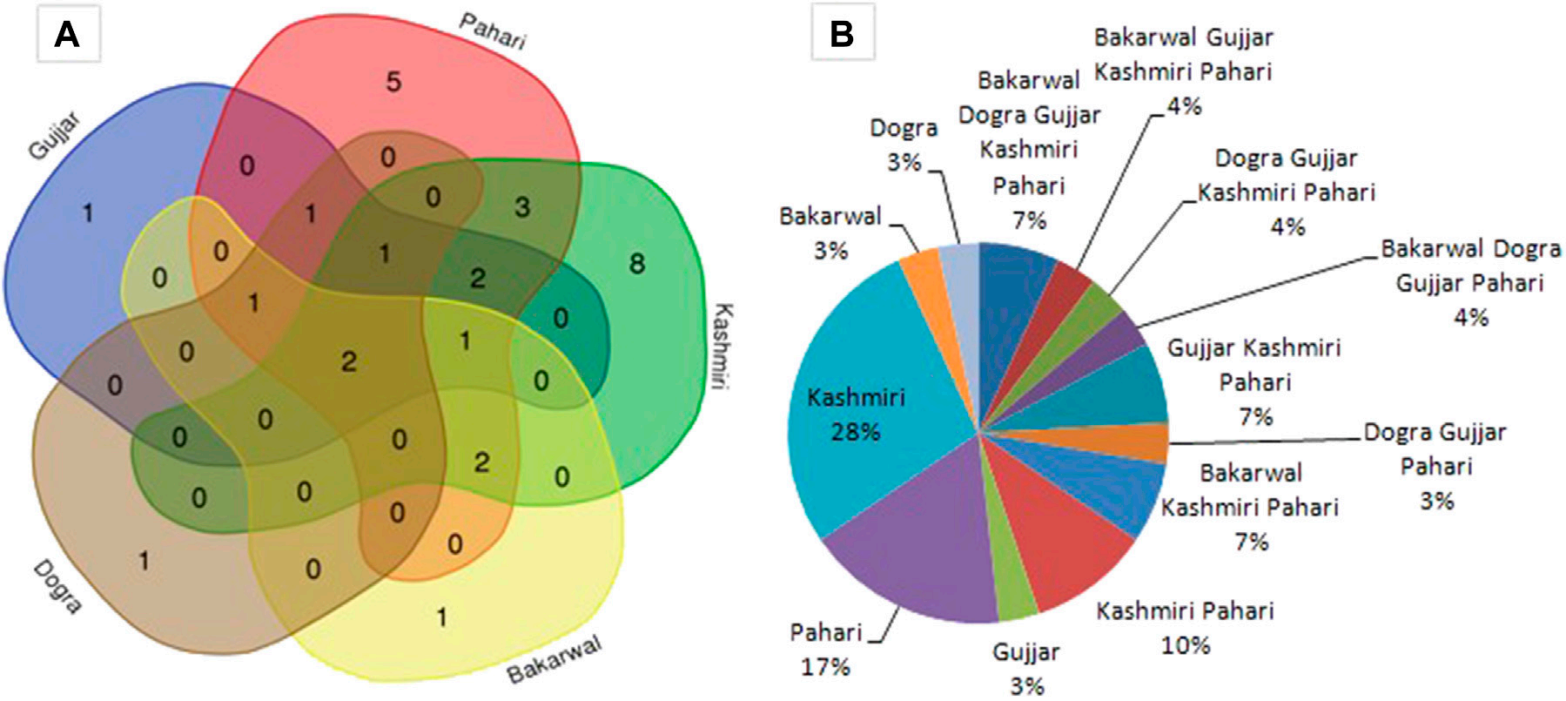
**(A)** Cross cultural use of documented species in diverse groups of Jammu and Kashmir; **(B)** percentage of similarity between ethnic groups.

The Venn diagram (Figure 4) shows that eight species (*Anopheles lindesayi*, *Macrovipera lebetinus*, *Neotibicen canicularis*, *Oxya japonica*, *Pangshura tecta*, *Stenamma kashmirense*, *Fejervarya limnocharis*, and *Apis florea*) were idiosyncratic to Kashmiri, followed by five idiosyncratic species (*Hoplobatrachus tigerinus*, *Pantala flavescens*, *Drawida japonica*, *Prionopelta kraepelini*, and *Daboia russelii*) to Pahari. *Platyceps ventromaculatus*, *Araneus trifolium*, and *Plecoptera reflexa* were unique to Gujjar, Bakarwal, and Dogra, respectively. The common usage of species (idiosyncratic) by the Kashmiri population is due to the socio-cultural dominance which can also be observed by the fact that other ethnic communities (Gujjar, Pahari, Dogra, and Bakarwal) sometimes call various documented species by Kashmiri local names; for instance, Kashmiri call *Neoscona theisi* as Zalur, and other communities like Gujjar, Bakarwal, and Paharia also use the same common name. Similarly, *Apis mellifera* is known as *Manchar* in Kashmiri, and all other selected ethnic groups use the same common name.

A cross-cultural comparison of the documented fauna showed that only two species (*Apis dorsata* and *Apis mellifera*) overlapped between the five ethnic communities (Figure 4). This can be explained by the fact that *Apis mellifera* is kept for producing honey by all ethnic groups and that *Apis dorsata* is a wild bee, mostly found in forests, believed to be more effective in treating health disorders than any other species. All local ethnic groups also believe that the honey from *Apis dorsata* has the potential to increase life span. The variations in the uses of the reported species could be referred to the wide array of socio-cultural differences among the studied ethnic groups which are located at different geographical locations in the study area. The use of the reported species was also shaped by the religious affiliations; for instance, we found that the Dogra were reluctant to use animals due to their religion (Hinduism) which does not allow them to kill animals. Only six species (*Plecoptera reflexa*, *Apis cerana*, *Apis mellifera*, *Apis dorsata*, *Pediculus humanus capitis*, and *Naja*) were listed by them, none of which were killed for ethnousage (Table. 2). Hassan et al. (1994) also reported the influence of religious affiliation in Dogra while investigating the traditional usage of mammals (wild and domestic) across different ethnic groups in J&K.

**FIGURE 5:**
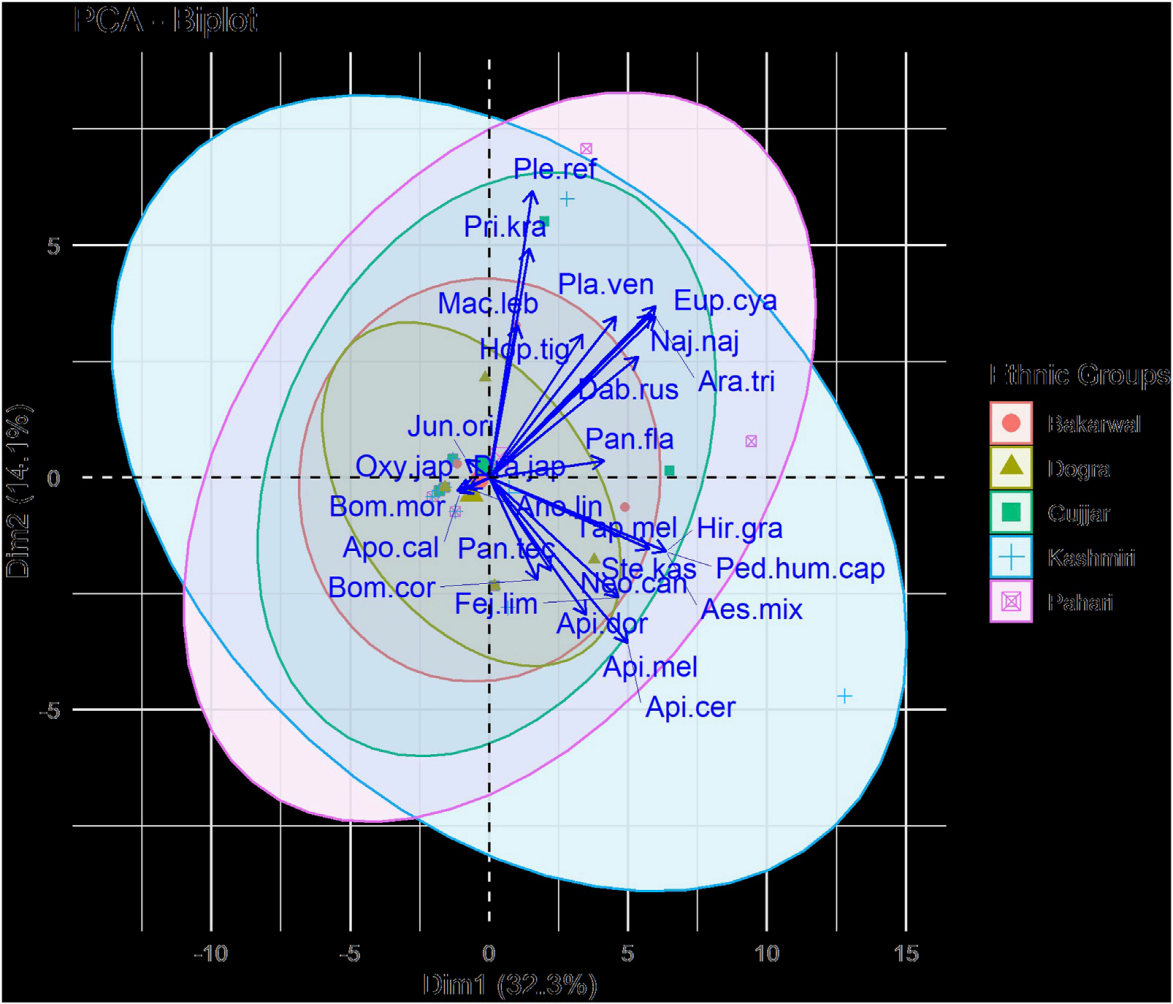
PCA diagram representing clustering of species among ethical groups.

**FIGURE 6:**
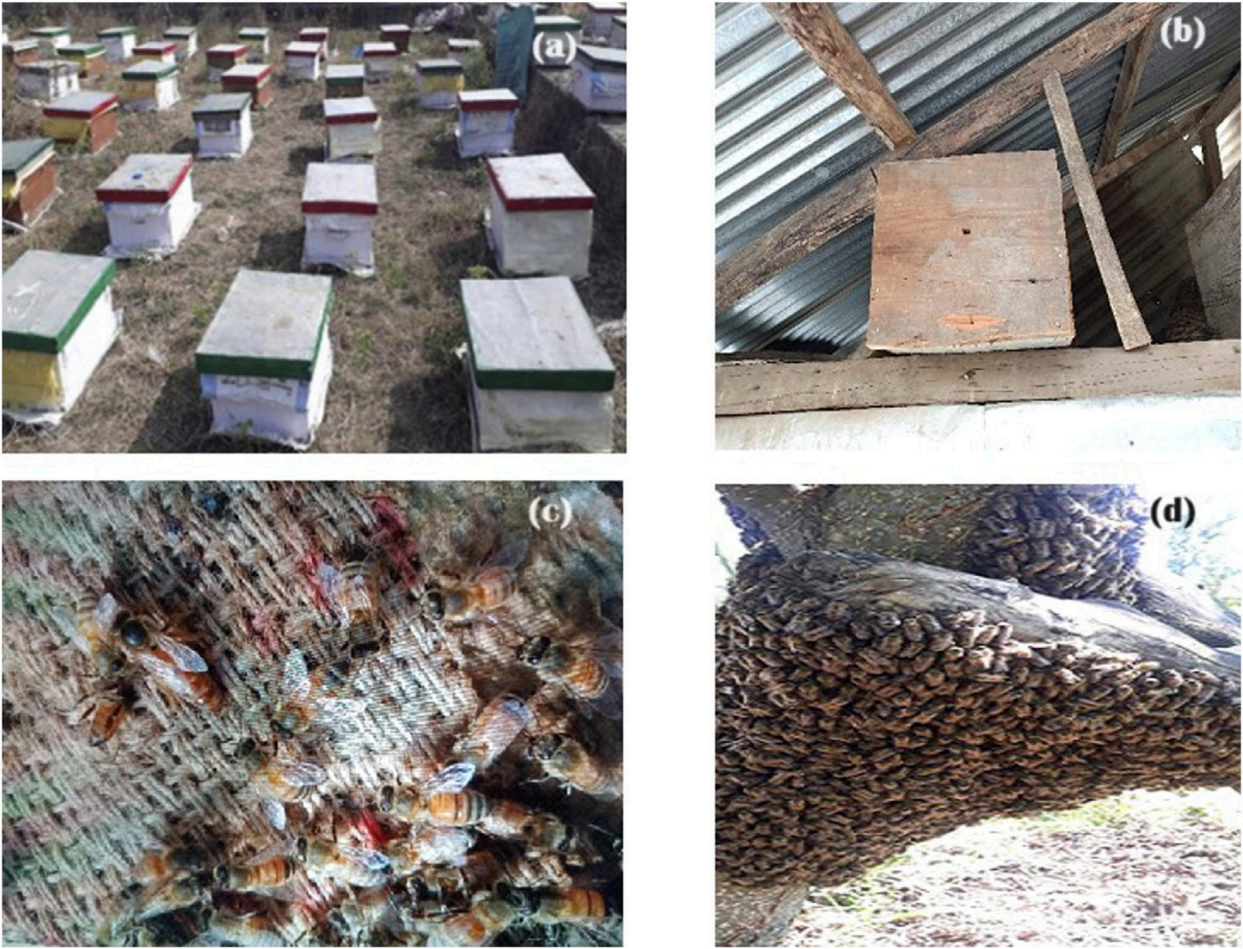
Different bees and bee related objects sited during field study in J&K, India **(A)** Apiary **(B)** Traditional bee hive **(C)** *Apis cerana* **(D)** *Apis mellifera*.

PCA showed considerable variation in the selected ethnic groups, and specific species were more closely related to a particular ethical group (Figures 5, 6). For example, PC1 (36.9%) and PC2 (16.4%) of species distribution in the biplot included species belonging to Kashmir and Pahari which grouped on two separate sides of the PCA, while species belonging to Gujjar, Bakarwal, and Dogra formed a separate cluster based on the presence or absence of species (Figure 5). Our findings are in accordance with Sajem and Gosai (2006), who reported the diversity of use patterns of fauna across different ethical tribes in Assam, India. Similarly, García del Valle et al. (2015) reported on the use of fauna across two different ethnic communities in Mexico. Hassan et al. (2021) documented the use of animal fauna by different ethnic groups in Jammu and Kashmir, India.

To understand the relation between species and their uses, use value (UV) was used. A complete list of the UV of the documented species across selected ethnic groups inhabiting the different parts of J&K is provided in Table 2. The highest UV was calculated for *Apis cerana* (0.40), followed by *Apis mellifera* (0.35), *Bombyx mori* (0.33), *Apis florea* (0.28), *Apis dorsata* (0.26), *Naja* (0.24), *Hoplobatrachus tigerinus* (UV = 0.24), and *Euphlyctis cyanophlyctis* (0.22). The high UV of the documented fauna can be attributed to livelihood generation and ethnomedicinal and magico-religious usage. Other studies (from other parts of the Himalayas) that are in accordance with our results are Altaf et al. (Kakati et al., 2006) Mussarat et al. (Das, 2015); Kakati et al. (Wells et al., 1993).

### 3.5. Religious and ritual usage

Different animal species are often important to different communities with respect to religion (Albuquerque et al., 2020). In the present study, we documented that a variety of species were linked to religious identity. Among Muslims, spiders are treated as sacred and believed to have protected the prophet Mohammad from enemies by covering the cave he inhabited with a web. Bees are described as a source of healing in the Quran. Similarly, in Hinduism, the goddess Bhramari is regarded as the goddess of bees, so people treat honey as an elixir. *Naja* (Indian cobra) is also treated as sacred in Hinduism as it is believed to be present around the neck of Lord Shiva who, according to Hindu mythology, is the destroyer (Vanashak) of the world.

In Kashmir, species such as *Macrovipera lebetinus* (blunt-nosed viper) and *Daboia russelii* (Doboia), and in Jammu, *Prionopelta kraepelini* (ant) and *Hoplobatrachus tigerinus* (Indus valley bullfrog), were used for supernatural purposes (magic). People who had knowledge of “supernatural” were very famous and generally known as “*Pirs*” in Kashmir and “*Babas*” in Jammu.

### 3.6. Myths across communities about invertebrates and herptiles

Across the region (J&K), during field surveys, various common myths were transcribed, and locals believe in them very strongly. Altaf et al. (Jamir and Lal, 2005) reported the variety of mythological perspectives about fauna species from Punjab, Pakistan.

The highest number of myths was recorded from the Kashmiri population, followed by Dogra.

• It is a common assumption of the Gujjar, Pahari, and Dogra that *Naja* (Indian cobra) can change into human shape after 100 years.
• A majority of the elderly people in Dogra believe that a stone called *mandi* found on the head of the Indian cobra has the potential to cure snake bites and fulfill any wish.
• In Pahari and Dogra, seeing snake biting in dreams is believed to be a sign that an enemy will win against you.
• Among the Kashmiri, if *Junonia orithya* (blue pansy) is found inside the home, it is believed to bring good fortune and wealth.
• Among the Kashmiri, *Anopheles lindesayi* (mosquito) is believed to help in paddy ripening.
• As per Kashmiri tradition, webs of spiders kept for a long time in homes are believed to bring misfortune.
• The Kashmiri believe that if *Pediculus humanus capitis* (head louse) is found in large numbers inside the bedroom, it indicates the arrival of misfortune.
• Among the Dogra, *Plecoptera reflexa* is believed to be a virtual form of a demon.

### 3.7. Anthroponyms and societal nomenclature

Across different parts of the world, anthroponyms and toponyms are common in many cultures (Chiwanga and Mkiramweni, 2019). This study tried to establish the relation between the existing meaning and use of the names of species across the selected ethnic groups (Table 3) which are in line with the study carried out by Chiwanga and Mkiramweni (2019) from Tanzania, who reported the avian fauna anthroponyms across different ethnic groups.

**TABLE 3:** Anthroponyms and societal nomenclature in Jammu and Kashmir, India.

S.No	Local name	Meaning attached to the local species name in selected ethnic groups				
		Kashmiri	Gujjar	Bakarwal	Pahari	Dogra
1	Qachve	The name (Qachve) is taken from the *Pangshura tecta* and represents a sluggish walker by denoting poor pace	-------------------	-------------------	-------------------	-------------------
2	Sap	-------------------	The term Sap, which comes from the *Platyceps ventromaculatus*, represents someone who will hurt you even after you have taken care of him	-------------------	-------------------	-------------------
3	Naag	-------------------	-------------------	-------------------	-------------------	From the species "*Naja naja*," the word "Naag" is derived. Having envy is referred to as "naag"
4	Draik	The name (Draik) is derived from *Hirudinaria granulosa*, given to the person who is acting as a parasite, using other people’s resources. “Draik” means one who sucks blood	-------------------	-------------------	The name (Draik) is derived from *Hirudinaria granulosa*, which is used to describe someone who uses the resources of others in a parasitic manner. “Draik” refers to a bloodsucker. “Draik” means one who sucks blood	-------------------
5	Khut Gunas	The term (Khut Gunas) comes from *Macrovipera lebetinus*, which is given to those who frequently act violently. “Khut” denotes quick action, while “Gunus” denotes a long, cylindrical body	-------------------	-------------------	-------------------	-------------------
6	Dav Rai	The name (Dav Rai) is derived from *Tapinoma melanocephalum*, given to the person who harms silently. “Dav” means giant, and “Rai” means who walks silently	-------------------	-------------------	-------------------	-------------------
7	Maduu	The name (Maduu) is derived from *Apis dorsata*, given to the person who cares and gets angry for small things. “Maduu” means showing both love and anger	-------------------	-------------------	-------------------	-------------------
8	Cheen	The name (Cheen) is derived from *Cicadatra acberi*, given to those newborn babies who often throw tantrums. “Cheen” means someone who makes a loud prickly noise	-------------------	-------------------	-------------------	-------------------
9	Zovaan	-------------------	-------------------	Name for divorced daughters who live with their parents. Zovaan is derived from *Pediculus humanus capitis*	-------------------	-------------------

### 3.8. Use of documented fauna for livelihood

**FIGURE 7:**
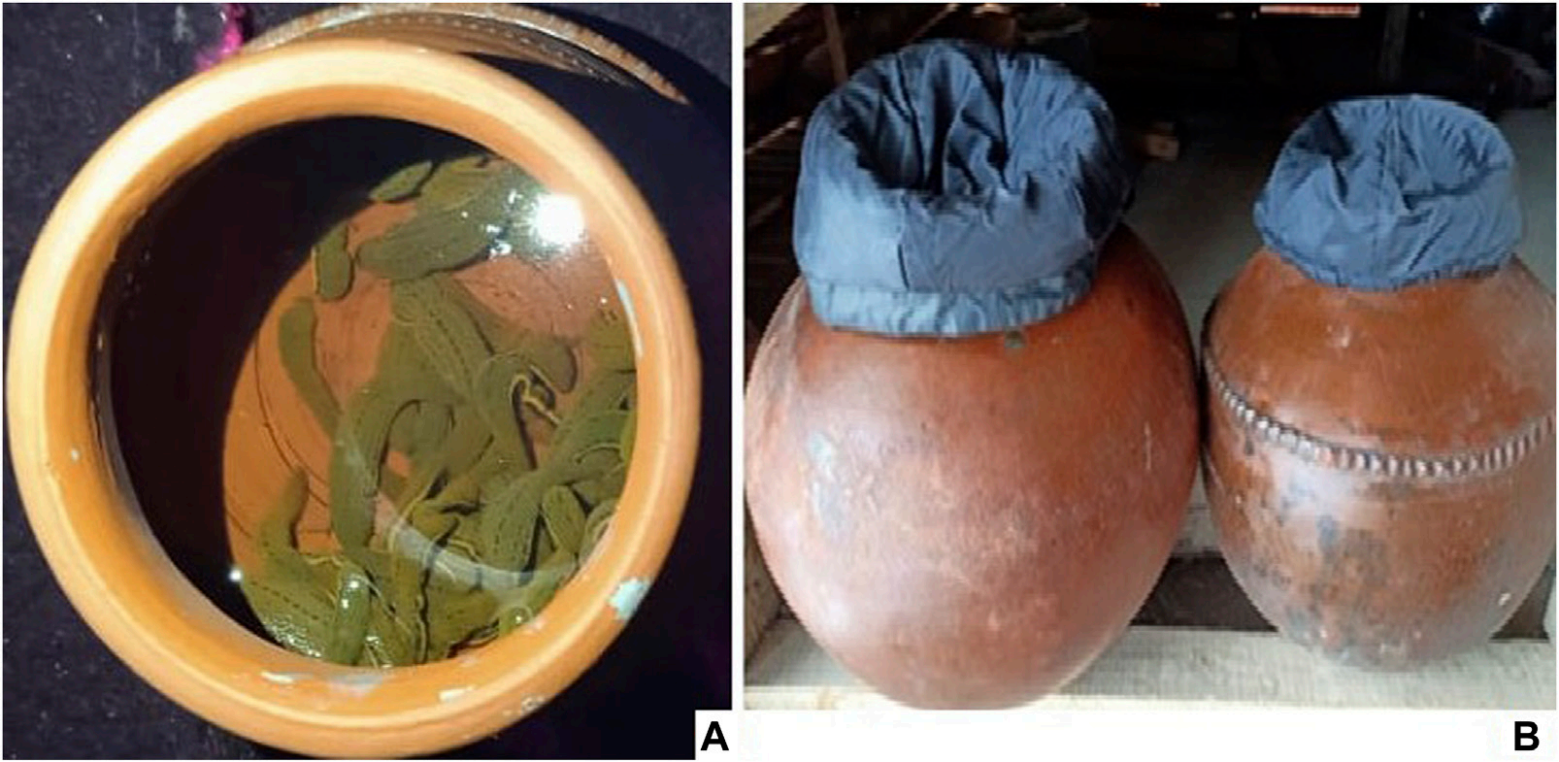
**(A)** Hirudinaria medicinalis **(B)** Traditionally pot used to keep medicinal leeches in J&K, India.

tIn J&K, the livelihood of the majority of the population revolves around agriculture and allied sectors (Table 1). However, some ethnozoological practices are part of the local system for livelihood generation. For instance, *Apis cerana* and *Apis mellifera* (photo plates Figures 6C,D) are used by locals to produce honey locally, called *Maanch/Shahed* for commercial purposes. The traditional hives called *dadoor* (photo plate Figure 6B) are made from deodar wood (*Cedrus deodara*). Respondents believed that deodar wood has the potential to protect the hive from a variety of diseases. All ethnic groups use this traditional hive, and ethnic groups such as Kashmiri and Pahari also use white mulberry wood (*Morus alba*) due to the belief that honey produced in mulberry wood hives would be richer in medicinal value. Some younger people also used to develop apiaries (photo plate Figure 6A) as their primary source of income but following modern methods and techniques. *Hirudinaria granulosa* locally called *Draik* was used for bloodletting by different people to generate income (Figures 7A,B). *Bombyx mori* was reared to obtain silk which is an effective livelihood source of income in the region. Olana and Demrew (2018) reported that *Apis* species were very important with respect to livelihood generation and the improvement of the local communities. Bhatia et al. (2011) reported on improving the livelihood of tribal people by adopting silkworm rearing in Chhattisgarh, India. Tulu et al. (2018) reported the usage of *Hirudinaria granulosa* as an alternative source of income in southwestern Ethiopia.

## 4 Conclusion

The present study is the first of its kind in the region (J&K) to evaluate the ethnozoological and cross-cultural usage of invertebrates and herpetofauna species. In pharmacological research, invertebrates and herptiles might be an interesting resource; however, the said medicinal fauna has received little attention so far, especially in the western Himalayas. In this regard, bio profiling of the species used in ethnomedicine might pave the way for drug development. The usage of faunal resources for ethnomedicinal uses also possesses a vital cultural dimension, as local people depend on the documented species (invertebrates and herptiles) for a variety of uses; further detailed research is needed to elucidate the multicultural association with the fauna species in the region, which in turn can help the stakeholders draft the policies for the development of local ethnic people. Traditional knowledge is mainly held by the elderly, which can be attributed to their lifelong observation and inheritance of traditional knowledge from their forefathers. The study can help by providing a baseline to draft future research projects and will make a further assessment to understand the interaction between local fauna and the ethnic groups.

## Data Availability

The original contributions presented in the study are included in the article/Supplementary Material; further inquiries can be directed to the corresponding authors.
