# Corrigendum: Invertebrates and herptiles for livelihoods—ethnozoological use among different ethnic communities in Jammu and Kashmir (Indian Himalayas)

**DOI:** 10.3389/fphar.2024.1426320

**Published:** 2024-05-14

**Authors:** Musheerul Hassan, Shiekh Marifatul Haq, Muhammad Shoaib Amjad, Riyaz Ahmad, Rainer W. Bussmann, José Manuel Pérez de la Lastra

**Affiliations:** ^1^ Clybay Research Private Limited, Bangalore, India; ^2^ Department of Ethnobotany, Institute of Botany, Ilia State University, Tbilisi, Georgia; ^3^ Department of Botany, Women University of Azad Jammu & Kashmir, Bagh, Pakistan; ^4^ Birmingham Institute of Forest Research, University of Birmingham, Birmingham, United Kingdom; ^5^ National Center for Wildlife, Riyadh, Saudi Arabia; ^6^ State Museum for Natural History, Karlsruhe, Germany; ^7^ Biotechnology of Macromolecules Research Group, Instituto de Productos Naturales y Agrobiología (IPNACSIC), San Cristóbal dela Laguna, Spain

**Keywords:** cross-culture, ethnozoology, medicinal animals, livelihood, Kashmir

In the published article, there was an error in Figure 6 as published. The figure included a version of 6c which was used as an informant prop for the participants of the study and was erroneously included in the final version of the figure. The corrected Figure 6 and its caption appear below.

**FIGURE 6:**
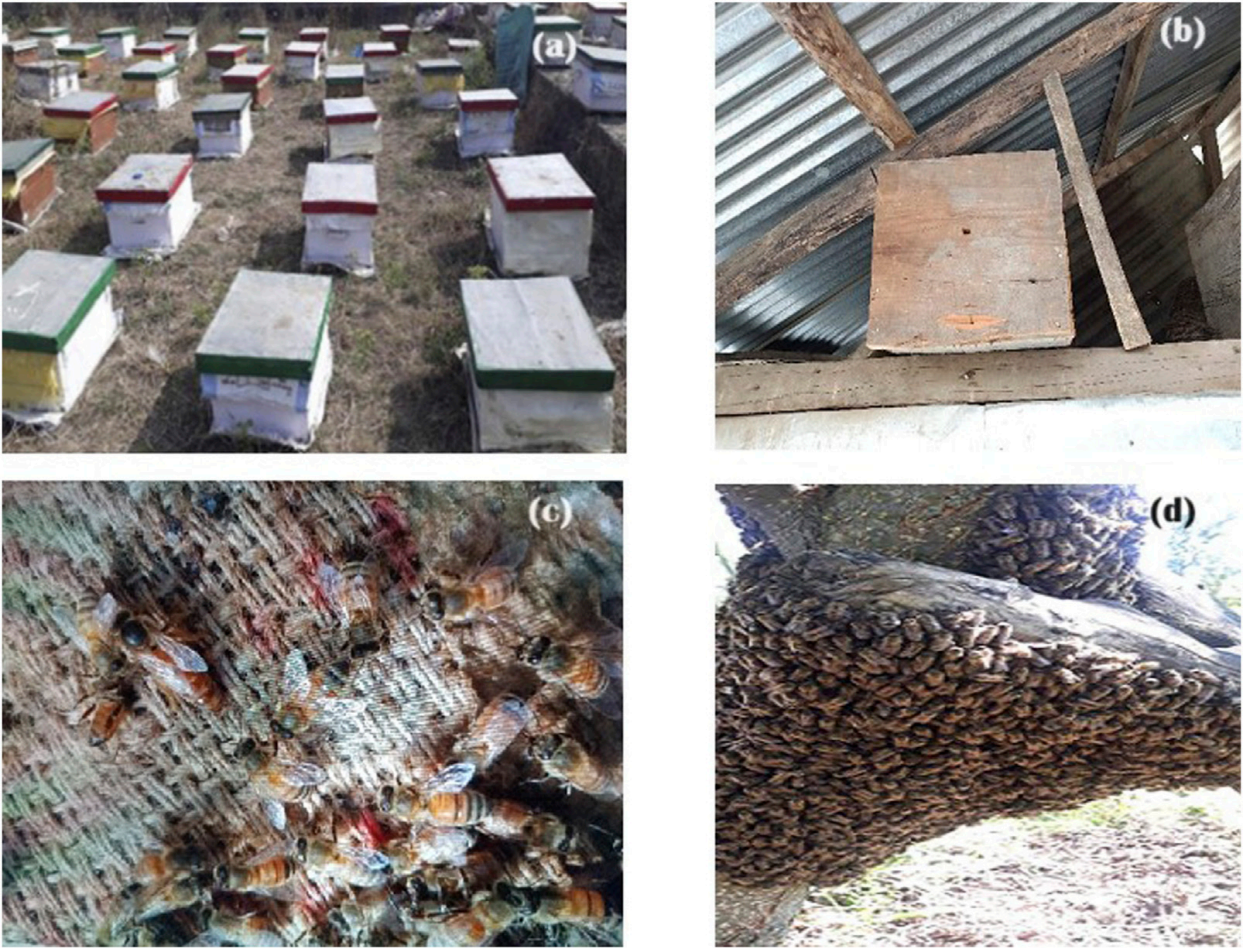
Different bees and bee related objects sited during field study in J&K, India **(A)** Apiary **(B)** Traditional bee hive **(C)** *Apis cerana* **(D)** *Apis mellifera*.

The authors apologize for this error and state that this does not change the scientific conclusions of the article in any way. The original article has been updated.

